# Autophagy and Nutrients Management in Plants

**DOI:** 10.3390/cells8111426

**Published:** 2019-11-12

**Authors:** Qinwu Chen, Daiki Shinozaki, Jie Luo, Mathieu Pottier, Marien Havé, Anne Marmagne, Michèle Reisdorf-Cren, Fabien Chardon, Sébastien Thomine, Kohki Yoshimoto, Céline Masclaux-Daubresse

**Affiliations:** 1 Institut Jean-Pierre Bourgin, INRA, AgroParisTech, CNRS, Université Paris-Saclay, 78000 Versailles, France; qinwu.chen@inra.fr (Q.C.); luojie654@163.com (J.L.); Marien.Av@Hotmail.Fr (M.H.); anne.marmagne@inra.fr (A.M.); michele.cren-reisdorf@inra.fr (M.R.-C.); fabien.chardon@inra.fr (F.C.); 2 Department of Life Science, School of Agriculture, Meiji University, Kawasaki, Kanagawa 214-8571, Japan; cf180412@meiji.ac.jp (D.S.); kohki_yoshimoto@meiji.ac.jp (K.Y.); 3 Life Science Program, Graduate School of Agriculture, Meiji University, Kawasaki, Kanagawa 214-8571, Japan; 4 Institut de Biologie Intégrative de la Cellule, CNRS, Avenue de la Terrasse, 91198 Gif-sur-Yvette, France; mpottier@uliege.be (M.P.); Sebastien.THOMINE@i2bc.paris-saclay.fr (S.T.)

**Keywords:** nitrogen use efficiency, leaf senescence, iron, zinc, nitrogen remobilization, plant proteases

## Abstract

Nutrient recycling and mobilization from organ to organ all along the plant lifespan is essential for plant survival under changing environments. Nutrient remobilization to the seeds is also essential for good seed production. In this review, we summarize the recent advances made to understand how plants manage nutrient remobilization from senescing organs to sink tissues and what is the contribution of autophagy in this process. Plant engineering manipulating autophagy for better yield and plant tolerance to stresses will be presented.

## 1. Introduction

Intracellular recycling plays an essential role in the proper control of cellular events, such as modulating the levels of key regulators, and more importantly, as the main housekeeper that removes cellular debris and replenishes essential nutrients to support new growth [1,2]. The best-studied and understood recycling system in plants is the ubiquitin-proteasome pathway in which proteins are ligated with a poly-ubiquitin chain to serve as effective substrates for cleavage by the 26S proteasome [3]. However, the selective degradation of this system is limited to some individually damaged or short-lived (regulatory) proteins and seems insufficient in bulk protein degradation during leaf senescence. Then, plants employ the autophagy pathway for vacuolar bulk turnover of cytoplasmic components. Autophagy entails the encapsulation of unwanted cytosolic materials within specialized autophagic vesicles, which are subsequently delivered to the vacuole for proteolysis or hydrolysis [4]. 

**Figure 1:**
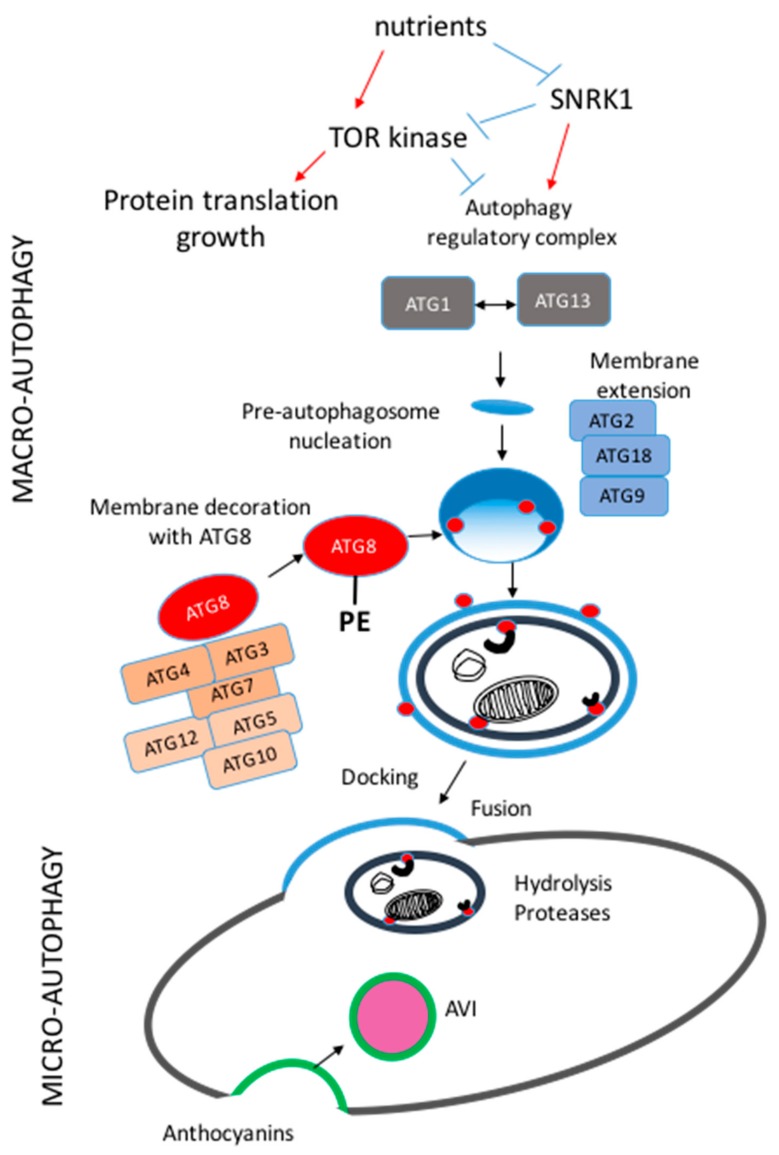
Schematic representation of macro-autophagy and micro-autophagy pathways in plants. Nutrient availability controls the TOR (target of rapamycin) kinase activity that, in turn, regulates post-transcriptionally maro-autophagy through the phosphorylation of ATG1 and ATG13 (autophagy proteins 1 and 13). After nucleation of the pre-autophagosome structures, the autophagy ATG9, ATG18, and ATG2 proteins (in blue) are involved in the expansion of the membrane of the autophagosome. Several autophagy (ATG) proteins (in orange) involved in the conjugation of ATG8 to phosphatidyl-ethanolamine facilitate ATG8 anchorage to the membrane of the pre-autophagosome and per se autophagosome formation and enclosure. The ATG8-interacting motifs facilitate the capture of cargoes to be driven to the central vacuole for degradation. Micro-autophagy consists of the invagination of the tonoplast and participates in the formation of anthocyanin vacuole inclusions (AVI).

Three distinct types of autophagy, micro-, macro- and mega-autophagy, have been reported in plants. Micro-autophagy proceeds by the invagination of the tonoplast to trap cytoplasmic material congregated at the vacuole surface to create autophagic bodies within the vacuole (Figure 1). Such a micro-autophagy process is poorly described in plants. It was found that the transport of cytoplasmic anthocyanin aggregates into the vacuole is mediated by a process reminiscent of micro-autophagy [5,6,7]. Conversely, macro-autophagy is much better described. It involves double-membrane vesicles, named autophagosomes, that sequester cytosolic components [8]. After traffic to the lytic vacuoles, their outer membrane fuses with the tonoplast to release their contents (inner membrane plus cargoes) into the vacuolar lumen. Released bodies are then called autophagic bodies. Autophagic bodies containing luminal constituents are broken down by resident vacuolar hydrolases, and the products are exported back to the cytosol for reuse. While micro-autophagy decreases tonoplast membrane area, macro-autophagy provides new lipid material to the tonoplast. Thus, it is likely that these two types of autophagy may play opposite roles in tonoplast membrane homeostasis. Mega-autophagy involves the massive degradation of the cell at the final phase of developmentally programmed cell death (PCD) [9]. During this process, the permeabilization or rupture of tonoplast results in the release of large amounts of hydrolases into the cytoplasm, which completely degrades the cytoplasm and even the cell walls, leading ultimately to cell death. Mega-autophagy has been mainly described in the case of xylem formation in plants [10]. 

Of these three types, macro-autophagy is the best-characterized process and is considered as the major form and has received the most attention [11]. During leaf senescence, expression of several AuTophaGy-related (*ATG*) genes encoding key components for autophagosome formation is increased [12]. The suppression of these genes disrupts the normal development of autophagosomes and results in hypersensitivity to starvations as well as premature leaf senescence. This suggests that autophagy plays a key role in leaf senescence and nutrient recycling [13,14,15,16].

## 2. Molecular Machinery of Macro-Autophagy in Plants

Professor Yoshinori Ohsumi was awarded the Nobel Prize for Physiology or Medicine in 2016 for his discovery of the molecular basis of macro-autophagy (hereafter referred to as autophagy). Together with colleagues, he identified several *ATG* genes that participate in autophagic processes by yeast forward genetic experiments [17]. To date, more than 40 *ATG* genes have been identified in yeast, and the orthologs for most of them have been found in different plant species such as *Arabidopsis*, rice, wheat, maize, tobacco, barley, foxtail millet, and apple [12,13,18,19,20,21,22,23]. The functional analysis of these proteins reveals a canonical route for autophagy. Basically, the process of autophagy consists of the induction of the nucleation of pre-autophagosomal structures, membrane elongation, phagophore expansion, and then closure, trafficking, and delivery of the autophagosome to the vacuole, and finally breakdown of the autophagic membrane and its contents by hydrolases into the vacuole (Figure 1) [2]. The ATG1 and ATG13 proteins, together with two accessory proteins, ATG11 and ATG101, assemble into an active ATG1-ATG13 complex [24] that promotes the nucleation and expansion of a cup-shaped double-membrane (phagophore), which is thought to originate from the endoplasmic reticulum (ER) [25,26,27]. The transmembrane protein ATG9 recruits lipids for phagophore elongation, ATG2, and ATG18 proteins facilitate ATG9 cycling [16,26]. Another step involves phagophore decoration with phosphatidylinositol-3-phosphate (PI3P) generated by a class III complex containing the phosphatidylinositol-3-kinase (PI3K) encoded by vacuolar protein sorting 34 (VPS34), along with three core accessory subunits, ATG6, VPS38 or ATG14, and VPS15 [28,29]. Expansion and closure of the phagophore membranes require two ubiquitination-like systems. Ubiquitin-fold protein ATG8 is initially processed by a cysteine protease ATG4 to expose a C-terminal glycine [30,31], then conjugated to the lipid phosphatidylethanolamine (PE) by the conjugating enzyme ATG3 [15]. Another ubiquitin-fold protein ATG12 is conjugated to ATG5 by the conjugating enzyme ATG10 [32]. Both of the conjugation systems share a single ATP-dependent activating enzyme ATG7 [13]. The ATG12-ATG5 conjugate promotes the lipidation of ATG8 with PE and its anchorage into the phagophore membrane [33]. ATG8 decoration of the phagophore membrane facilitates the recruitment and seal of the cargoes inside the autophagosome. ATG4 is also needed to remove and recycle ATG8 from ATG8-PE lining the outer membrane [30], while the ATG8-PE adducts trapped on the autophagosome inner membrane are digested in the vacuole. The autophagosome then transports the cargoes to the vacuole by fusing the outer membrane with tonoplast, and the remaining single-membrane structure (autophagic body) is released inside the vacuole for degradation by proteases and hydrolases. The digested products are then exported from the vacuole for recycling. 

The TOR (target of rapamycin) protein kinase is a master player in sensing the nutrient status of eukaryotic cells. TOR orchestrates cell homeostasis in fine crosstalk with several other players, among which its LST8 and RapTOR partners and SnRK1 (Snf1-related protein kinase 1) kinase [34]. TOR is a well-known positive regulator of ribosome protein synthesis and of translation and a negative post-translation regulator of autophagy under nutrient-rich conditions. It dampens autophagy at the post-translational level by hyper-phosphorylating ATG13, which prevents its association with ATG1. Under nutrient-limiting conditions, the inactivation of TOR leads to rapid de-phosphorylation of ATG13, allowing it to bind ATG1. The TOR kinase also plays a role in regulating the transcription of genes. It activates genes involved in anabolic processes that are essential for rapid growth like amino acid, lipid, and nucleotide synthesis and the oxidative pentose phosphate pathway and represses genes mediating the degradation of proteins, amino acids, lipids and xenobiotic, and autophagy regulation [35]. On the contrary, SnRK1 is a positive regulator of autophagy in Arabidopsis. The KIN10 SnRK1 alpha catalytic subunit is necessary for the activation of autophagy under energy depleted conditions and in response to many other abiotic stresses. SnRK1 can control autophagy through both TOR-independent and TOR-dependent pathways, depending on stresses [36]. Autophagy genes and autophagic activity are then strongly induced by nitrogen and carbon limitation in several plant species, as well as by many other stresses as reviewed by Tang and Bassham [37]. 

## 3. Selective Macro-Autophagy

Although autophagy was originally considered as an unrestricted bulk degradation of cytoplasm compounds, recent studies reveal that various routes for selective autophagy exist. Selective autophagy can specifically degrade appropriate cargoes by engaging a wide array of receptors or adaptor proteins that tether the cargoes and also interact with ATG8 [38,39,40]. The interaction between autophagic receptors and ATG8 is mediated by the presence of the ATG8-interacting motif (AIM) in each receptor [41]. Recently, a new binding site for autophagy adaptors and receptors was discovered on ATG8. This site engages ubiquitin-interacting motif (UIM)-like sequences rather than the canonical AIM for high-affinity binding to a new class of ATG8 interactors [42]. As ATG8 decorates and controls phagophore membrane expansion, its abundance determines the size of the autophagosome [43]. In this way, autophagosomes may undergo drastic membrane expansion and develop into multiple sizes to efficiently and selectively sequester specific cargoes, including protein aggregates, mitochondria, peroxisomes, chloroplasts, proteasome, ribosomes, endoplasmic reticulum, invading pathogens, and other components in plant cells under specific conditions.

Several forms of selective autophagy have been reported in plants as chlorophagy (degradation of chloroplasts), reticulophagy (degradation of endoplasmic reticulum), mitophagy (degradation of mitochondria), pexophagy (degradation of peroxisomes), proteaphagy (degradation of proteasomes), ribophagy (degradation of ribosomes), aggrephagy (degradation of intracellular protein aggregates), xenophagy (degradation of intracellular pathogens), degradation of the pre-autophagosomal structure, degradation of TSPO (tryptophan-rich sensory protein), and the degradation of brassinosteroid-responsive transcription factor BES1 [2,44,45]. Selective autophagy certainly allows fine organelle quality control and also to the removal of specific cellular waste. At the same time, specific autophagy provides cargoes to degradation pathways performing hydrolysis and proteolysis inside the vacuole lumen; it facilitates the release of metabolites that contribute to nutrient recycling. Whether some specific autophagy pathways could be more related to the recycling of specific nutrient, macro- or micro-elements is an interesting question that remains to be investigated.

In plants, the first selective autophagy receptor, named Joka2, was identified in tobacco. This NBR1 (neighbor of BRCA1 GENE 1) homolog was identified in yeast two-hybrid screen carried out to look for partners of the coiled-coil protein UP9C of unknown function that strongly over-accumulates under sulfur-deficiency [46]. Afterward, the *Arabidopsis* NBR1 homolog was characterized and shown to target ubiquitinated protein aggregates formed under stress conditions through a C-terminal ubiquitin-associated (UBA) domain [46,47]. Like *ATG* genes, it was shown that NBR1/Joka2 expression is enhanced under several nutrient starvations as C, N, and S limitations. Functional analyses using two nbr1 knockout mutants revealed that (i) NBR1 is important for plant tolerance to a large spectrum of abiotic stresses, like heat, oxidative, salt, and drought stresses, and (ii) there is an increased accumulation of ubiquitinated insoluble proteins in nbr1 mutants under heat stress [48,49]. However, unlike *atg5* and *atg7* mutants, nbr1 is not sensitive to darkness stress or necrotrophic pathogen attack, suggesting that NBR1 is involved in the selective degradation of denatured or damaged non-native proteins generated under high temperature conditions, but not in other “bulk” autophagy. Therefore, autophagy operates through distinct cargo recognition and delivery systems according to biological processes. NBR1 is involved in the selective degradation of denatured or damaged non-native proteins generated under high-temperature conditions but is not involved in other “bulk” autophagy. Interestingly, it was recently reported that NBR1 also specifically binds viral capsid protein and particles of the cauliflower mosaic virus (CaMV) in xenophagy to mediate their autophagic degradation, and thereby restricting the establishment of CaMV infection [50]. Similarly, Joka2/NBR1 mediated selective autophagy pathway contributes to the defense against *Phytophthora infestans*. The *Phytophthora infestans* effector protein PexRD54 recognizes potato ATG8CL (potato CL isoform of ATG8) through an AIM [51]. PexRD54 outcompetes binding of ATG8CL with the Joka2/NBR1 to counteract defense-related selective autophagy, thus possibly attenuating autophagic clearance for pathogen or plant proteins that negatively impact plant immunity [51,52]. Upon infection, ATG8CL/Joka2 labeled defense-related autophagosomes are diverted to the host-pathogen interface to restrict pathogen growth focally [52].

Subsequently, the ATI1/ATI2 ATG8-binding proteins were also characterized as autophagy receptors. ATI1 is located in ER-bodied and plastid-associated bodies in dark-induced leaves [53,54]. The plastid localized ATI1-bodies were also detected in senescing cells and shown to contain stroma proteins. While they likely play a role in chlorophagy, their role in N remobilization during senescence has not been reported so far. 

Another example of a specific autophagy adaptor is RPN10 (Proteasome polyubiquitin receptor 10). The proteasome subunit RPN10 was shown to mediate the autophagic degradation of the ubiquitinated 26S proteasomes, known as proteaphagy [55]. Upon stimulation by chemical or genetic inhibition of the proteasome, RPN10 simultaneously binds the ubiquitinated proteasome, via a ubiquitin-interacting motif (UIM), and to ATG8 through another UIM-related sequence that is distinct from the canonical AIM motif. In Arabidopsis, the inhibitor-induced proteaphagy was blocked in mutants expressing an RPN10 truncation that removed the C-terminal region containing these UIMs. 

In addition to specifically eliminating macromolecular complexes, organelles, and pathogens, selective autophagy can also scavenge individual proteins. For example, TSPO (tryptophan-rich sensory protein) is involved in binding and eliminating highly reactive porphyrin molecules through autophagy by interacting with ATG8 proteins via a conserved AIM motif [56]. A more recent study proposed another role for TSPO to control water transport activity by interacting with and facilitating the autophagic degradation of a variety of aquaporins present in the tonoplast and the plasma membrane during abiotic stress conditions [57].

## 4. Nutrient Remobilization after Organelle and Protein Degradation in Senescing Leaves

Nitrogen is quantitatively the most important mineral nutrient for plant growth. The use of nitrogen by plants involves several steps, including uptake, assimilation, translocation, recycling, and remobilization [58]. Plants are static and cannot escape from the multitude of abiotic and biotic stress conditions occurring during their growth period. To deal with these environmental stresses and survive in the fluctuating environment, plants senesce leaves to massively remobilize phloem-mobile nutrients and energy from senescing leaves to developing tissues and storage organs. This way, plants can save and efficiently utilize the limited nutrients and energy for defense, growth, and reproduction [59]. Efficient nitrogen remobilization, thus increases the competitiveness of plants, especially under nitrogen limiting conditions. For agriculture, high nitrogen remobilization efficiency is interesting as it can reduce the need for nitrogen (N) fertilization, which represents a substantial cost of agricultural production and often causes environmental pollution. In crops, post-anthesis nitrogen remobilization during seed maturation is highly correlated to grain yield and quality [60]. In small-grained cereals like wheat and rice, up to 90% of the grain nitrogen content is remobilized from the vegetative plant parts, while the proportion in maize is approximately 35–55% [61].

Once senescence is initiated, carbon and nitrogen primary assimilations are progressively replaced by recycling from the catabolism of macromolecules such as proteins and nucleic acids. Up to 75% of the total mesophyll cellular nitrogen is localized in the chloroplasts [62]. The breakdown and recycling of these considerable nitrogen resources depend on three distinct chloroplast degradation pathways that rely on macro-autophagy, senescence-associated-vacuoles and Chloroplast Vesiculation (CV) pathways (see [63] and [2], for reviews). Up to now, although detailed knowledge concerning interactions and relationships between these three chloroplast degradation pathways remains insufficient, the cysteine proteases localized in the vacuole appear to play a particularly important role in all these processes as they proceed during the last steps of the macromolecule breakdown in the vacuole. Cysteine proteases as SAG12 (senescence associated gene 12), Cathepsin B3 (CATHB3), Responsive-to-desiccation 21A (RD21A), *Arabidopsis* aleurain-like protease (AALP) and Vacuolar Processing Enzymes (VPEs) are amongst the most highly overexpressed senescence-related proteases [64,65].

**Figure 2:**
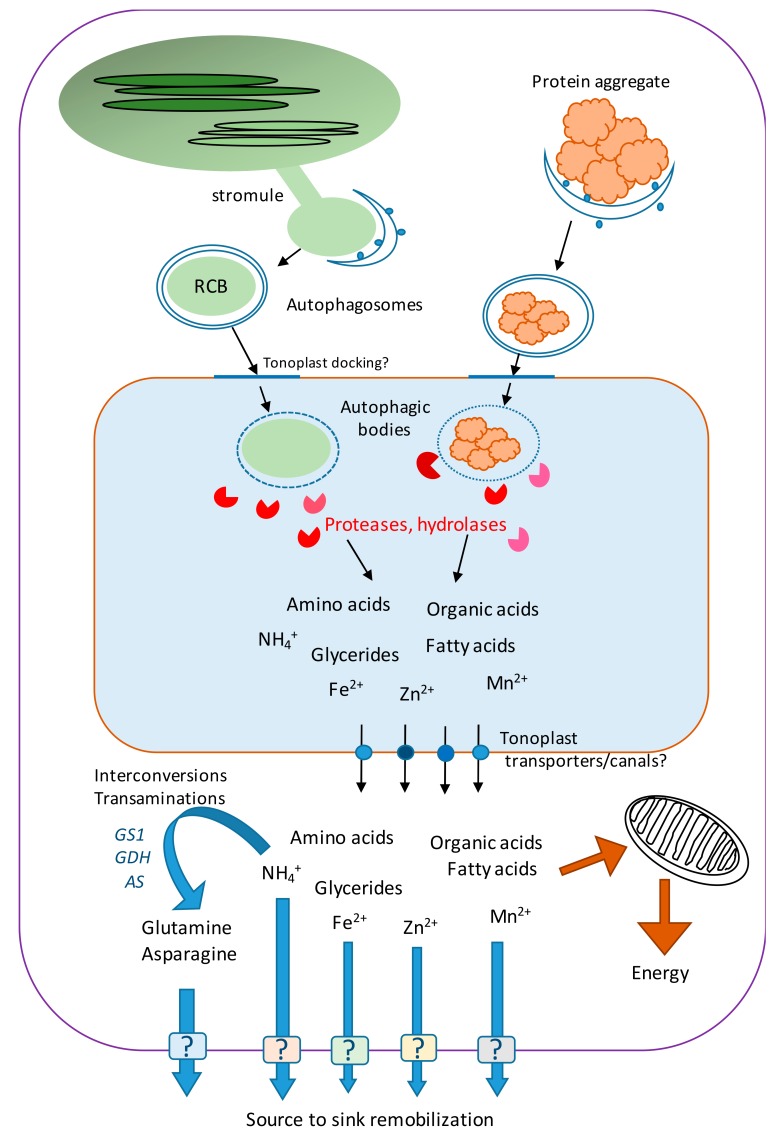
Schematic representation of the different steps of nutrient recycling in plant cells. Chloroplast material and unwanted cytoplasmic material are driven to the central vacuole for degradation through the macro-autophagy pathway. Once delivered to the vacuole lumen, autophagic bodies (inner membrane of autophagosome and cargoes) are degraded by the resident proteases and hydrolases. The nutrients released are exported to the cytosol and using transporters or canals. Once inside the cytosol, nutrients are either used for cell metabolism or released out of the cell for source to sink translocation. Interconversions of amino-acids occur in the cytosol to produce the glutamine and asparagine forms that are preferentially used for long-distance transport in the phloem. Many black boxes remain to be explored, especially regarding the docking of autophagosomes to the tonoplast and the transport of nutrients out of the vacuole and further out of the cell (question marks).

Efforts made to understand nitrogen remobilization during leaf senescence have mainly focused on the biochemistry of the degradation of plastidial proteins (Figure 2). Originally observed by immuno-electron microscopy in the cytoplasm and vacuole of naturally senescing wheat leaf cells, the RuBisCo-containing bodies (RCBs) were proposed to be involved in RuBisCo degradation process outside of the chloroplasts [66]. These RCBs contained the large and small subunit of RuBisCo and other stromal proteins as the chloroplastic glutamine synthetase. However, RCB lacked chloroplast envelope or thylakoid components. Sometimes, RCBs were found to be surrounded by double membranes, which seem to be derived from the chloroplast envelope. Interestingly several observations presented RCBs in the cytoplasm closely bordered by kinds of bean-shaped vesicles that might be isolation membranes characteristic of the intermediate structures of autophagosomes (phagophores) [66,67]. RCBs were frequently visible at the early stages of leaf senescence when RuBisCo starts to decrease without prior chloroplast destruction or chlorophyll degradation, and it was proposed that the budding of RCBs from chloroplast stromules was a way to bring chloroplast material out of the organelle [68,69,70]. This material release may explain why during senescence, the size of chloroplasts (c.a. 10 µm) decreases to form gerontoplasts (c.a. 4 µm). 

The demonstration that autophagy plays a prominent role in RCB trafficking to the vacuole was provided using confocal microscopy to visualize stromal and ATG8 proteins tagged with different fluorescent probes. The authors showed that the release of RCBs inside the vacuole required functional autophagy and was absent in autophagy mutants such as *atg5* and *atg7*. Co-localization of autophagosomes and RCBs was also demonstrated, and moreover, it was also shown that shrunken gerontoplasts could be released inside the vacuole in an ATG4-dependent micro-autophagy pathway [71,72]. The elimination of membrane damaged chloroplast via micro-autophagy was further confirmed, and the role of macro-autophagy related membranes harboring GFP-ATG8 decorations in this process suggested [73].

## 5. Role of Autophagy in Nitrogen Recycling

**Figure 3:**
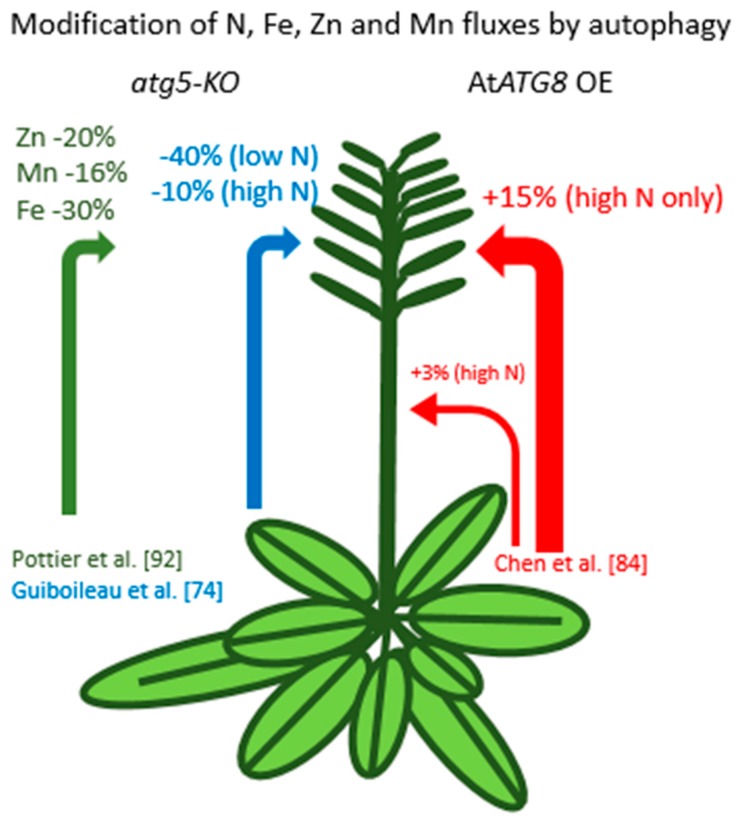
Modification of macro- and micronutrient fluxes in autophagy mutants and overexpressors in *Arabidopsis*. The green and blue arrows indicate the lack (as percentages) of micro and macro-nutrient allocation to the seeds in the *atg5* knock out (*atg5*-KO) mutant relative to wild type. The red arrows indicate the extra nitrogen remobilization measured in *ATG8 Arabidopsis* overexpressors, by comparison, to the control line under plethoric nitrate conditions.

As stated earlier, under normal conditions, autophagy operates at a basal level that constitutes housekeeping machinery and participates in cell homeostasis. Under nutrient starvation and during leaf senescence, autophagy activity is enhanced, and its role in nutrient recycling and remobilization at the whole plant level was suspected. The demonstration of the role of autophagy in nutrient recycling and mobilization from source to sinks was provided by Guiboileau et al. [74] (Figure 3). Monitoring ^15^N fluxes to the seeds after labeling *Arabidopsis* rosettes at the vegetative stage, Guiboileau et al. (2012) showed that N remobilization was markedly decreased in *ATG* mutants (*ATG18a* RNAi, *atg5*, and *atg9*) compared to wild type plants (WT). The decrease was more moderate when plants were grown under high nitrate than under low nitrate conditions, but still significant. Accordingly, the authors further found that *ATG* mutants accumulated more ammonium, amino acids (AA), proteins, and RNA in their rosette leaves than WT [75]. N remobilization was further evaluated using a similar ^15^N-labeling procedure in the *Zmatg12* maize mutants, which revealed that N remobilization to the kernels was also impaired in autophagy-deficient mutants [76].

The growth of *Zmatg12* mutants was most often arrested at the seedling stage, and adult plants showed enhanced leaf senescence and stunted ear development under nitrogen-starved conditions but not under high-N. Under nutrient-rich conditions, the seed yield of *Zmatg12* plants was much lower, and ^15^N reallocation into the seeds was twice less in *Zmatg12* was half of that in WT. The investigation conducted during the vegetative growth period on the rice autophagy-deficient mutant *Osatg7-1* suggested that N remobilization from senescent leaves to young leaves was suppressed [77]. Higher nitrogen content was retained in senescent leaves of *Osatg7-1* mutants as soluble protein, and RuBisCo concentrations were higher than that of WT. The reduction of nitrogen available for newly developing tissues in *Osatg7-1* likely led to its reduced leaf area, tillers, and photosynthetic capacity. Unfortunately, the male-sterile phenotype of *Osatg7-1* mutants prevented authors from examining the contribution of autophagy-mediated nitrogen remobilization from leaves to seeds during the reproductive growth period. 

Recently over-expression of autophagy genes was assessed in several plant species. Overexpressing *AtATG5* and *AtATG7* in *Arabidopsis* delayed senescence, improved seed production, and yield under certain conditions [78]. Similarly, several reports showed that overexpressing different *ATG8* genes from soybean or millet in *Arabidopsis* or rice was beneficial to plant performances, increasing tolerance to nitrogen starvations and to drought [79,80,81,82,83].

Using the same ^15^N labeling procedure as Guiboileau [74], Chen et al. [84] then showed that N remobilization of nitrogen from the rosette leaves to the seeds was improved in *Arabidopsis* plants overexpressing the *AtATG8a* or the *AtATG8g* gene. In these plants, N seed filling was increased, and the amount of nitrogen lost in dry remains decreased. Interestingly the N-remobilization performances of the *AtATG8a* and *AtATG8g* overexpressors were improved only when plants were grown under abundant nitrate supply but not when grown under N limited conditions. This can be explained by the fact that under N-limitation, autophagy activity is already enhanced, which cancels the benefit of stimulating *ATG8* expression through genetic transformation.

This demonstration of the beneficial effect on the nitrogen use efficiency of over-expressing *ATG8* was confirmed by Yu et al. [85]. The authors over-expressed the *OsATG8a* gene in rice and found that N% in seeds was increased while N% in dry remains was decreased, attesting better N remobilization to the seeds. Interestingly, like in *Arabidopsis*, the positive effect on plant performances was only observed under sufficient N supply but not under N-limitation.

## 6. Cross-Talk between Autophagy and Senescence-Related Cysteine Proteases

Although both autophagy and cysteine proteases are key players during leaf senescence, protein proteolysis, and nutrient recycling, as shown by recent publications from James et al. [86,87], the relationship between them remains largely unknown. It is admitted that proteins are not degraded inside the autophagosomes but rather transported by them to the lytic vacuoles where proteases and hydrolases operate. As said before, autophagy mutants are impaired in N remobilization, and they accumulate large amounts of proteins and amino acids in their rosette leaves. They also present significantly higher protease activities in their rosette leaves than the wild type, which supports the hypothesis that proteases and substrates cannot meet each other in autophagy mutants. In order to investigate the nature of the protease activities enhanced in autophagy mutants, Havé et al. [88] used shotgun proteomics to identify these proteases and specific probes to monitor their activity. Results showed that cysteine proteases accounted for the largest proportion (38%) of the over-abundant proteases in autophagy-deficient lines. Activity-based protein profiling (ABPP) analysis with DCG-04 revealed that activities of papain-like cysteine proteases (PLCPs) were higher in autophagy-defective plants grown under low-nitrate conditions. Further pull-down experiments using the DCG-04 biotinylated inhibitor of papain-like cysteine protease (PLCP), showed that the active PLCPs accumulated in autophagy mutants in low-nitrate condition were mainly SAG12, RD21A, CATHB3, and AALP. The western blots using RD21A, CATHB3, and SAG12 antibodies confirmed that both the mature and immature protease forms were accumulated in the mutant lines, suggesting that there was no defect in protease maturation or trafficking in the autophagy mutants. The specific over-accumulation of these PLCPs under low nitrate but not under high nitrate in autophagy mutants strongly suggested their involvement in N remobilization, and possibly provide alternative remobilization pathways to autophagy. Such hypotheses need to be confirmed by further investigations and biochemical studies using protease and autophagy double mutants. Havé et al. [88] also found that the CND41-like aspartate protease AED1 (apoplastic enhanced disease susceptibility-dependent 1) that have been described by Kato et al. [89] as one of the potential protease involved in RuBisCo degradation was also increased in autophagy mutant. Interestingly AED1 that was up-regulated in the senescing leaves of the sag12 mutants was also proposed to compensate for the absence of SAG12 activity for N remobilization [87].

## 7. Autophagy and Other Nutrients

Autophagy is likely involved in the recycling not only of proteins but also of membranes and other cell components that certainly contain micro-elements. It is well known that iron in the cell is mainly linked to ferritin and photosystem I, which are located in plants into the plastids in plants. In mammals, ferritin is degraded by NCOA4-mediated autophagy (ferritinophagy), which participates in controlling ferropoptosis and erythropoiesis [90,91]. Although the role of autophagy in the degradation of ferritin has not been demonstrated in the plant, it was found that the efficiency of iron (Fe) translocation from vegetative organs to the seeds is severely decreased in several autophagy mutants compared to wild type [92] (Figure 3). The authors confirmed the defect of iron translocation to the seeds in autophagy mutant using ^57^Fe labeling and tracing experiment. This study also showed that not only iron but also manganese (Mn) and zinc (Zn) are sequestered into the rosette leaves. Consistently the lower amounts of Zn and Mn in the seeds of autophagy mutants also suggest that their translocation is dependent on autophagy. This observation is consistent with the study of Eguchi et al. [93] that showed that autophagy is induced under Zn limitation conditions, and those autophagy-deficient mutants (*atg5*, *atg10*) exhibit early senescence phenotype under Zn limitation and limited growth recovery after Zn resupply.

More recently, the hypersensitivity of autophagy mutants to zinc limitation was confirmed (Shinozaki et al.; unpublished data). While zinc limitation induced autophagy in wild-type, it triggered the accumulation of proteins in autophagy mutants as a mark of autophagy defect. Interestingly, Zn-deficiency symptoms in *ATG* mutants recovered under low-light and iron-limited conditions, pointing out the role of Fenton-related oxidative stress in the response of plants to zinc deficiency. This also suggests that the induction of autophagy by zinc deficiency could be mediated by Fenton-generated hydroxy radicals. 

Inorganic phosphate-like nitrogen is one of the major macro-elements needed for plant growth. The recent paper from Naumann et al. [94] reveals that phosphate limitation stimulates autophagy in the root tips of *Arabidopsis*. Stimulation of autophagy by Pi deprivation was exacerbated in the pdr2 mutants, which are hypersensitive to Pi deficiency. PDR2 protein is located at the endoplasmic reticulum and was hypothesized to play a role in ER-quality control. Blocking ER stress in pdr2 mutant introducing ire1a mutation, or providing ER-stress inhibitors reduced autophagosome formation in response to Pi deprivation. This indicates that the ER-stress induced by low-Pi triggers autophagy in roots under low phosphate. The root growth of autophagy mutants was strongly reduced by Pi deprivation due to early root apical meristem differentiation that lowered meristem activity. When suppressing locally Pi sensing using phosphite application, meristem activity was restored in autophagy mutants. Decreasing iron concentration in the low Pi culture medium also restored apical meristem activity in autophagy mutant, suggesting by the way that iron would also play a role in the ER stress response to Pi deficiency, possibly through the production of reactive oxygen species.

Finally, it is well known that autophagy mutants are hypersensitive to carbon starvation, and the role of autophagy in recycling lipids from membranes and amino acids from proteins is likely important, upon carbon starvation, for energy production and certainly for providing building blocks for macromolecule synthesis [95,96]. Recently two papers reported the fundamental role of autophagy in the degradation of endomembrane and in lipid catabolism for peroxisome β-oxidation [97,98].

## 8. Conclusions

The results obtained from the studies of autophagy-defective mutants grown under various starvations clearly indicate the involvement of autophagy in the recycling and remobilization of nutrients at the whole plant level. The studies that increased autophagic activity through the over-expression of some *ATG* genes demonstrated that it could be a powerful approach to improve plant tolerance to starvations and nutrient remobilization from source to sinks. However, recent results enlighten the strong link between nutrient deprivation (N, S, Zn, and Pi), and oxidative stress and ER-stress [94,98]. This questions whether the ER, which is the source of lipid for autophagosome formation [27], could be a sensor of plant environmental stresses and an intermediate in autophagy induction.
